# Investigation of the adsorption behavior of BSA with tethered lipid layer-modified solid-state nanopores in a wide pH range†

**DOI:** 10.1039/c9ra00698b

**Published:** 2019-05-17

**Authors:** Haibing Tian, Wanyi Xie, Shixuan He, Daming Zhou, Shaoxi Fang, Liyuan Liang, Deqiang Wang

**Affiliations:** Chongqing Institute of Green and Intelligent Technology, Chinese Academy of Science Chongqing 400714 P. R. China dqwang@cigit.ac.cn; University of Chinese Academy of Sciences P. R. China

## Abstract

Nanopore technology was introduced for the study of the dynamic interactions between bovine serum albumin (BSA) and 1,2-dioleoyl-*sn*-glycero-3-phosphoethanolamine (DOPE) phospholipids based on a modified nanopore. The results reveal that the interaction mechanism between DOPE and BSA is affected by the pH of the subphase. Far above the BSA isoelectric point (pH > 7), a weaker hydrophobic interaction and stronger electrostatic repulsion exist between the DOPE and BSA molecules. At pH = 7, the BSA structure nearly does not change, and the interaction is weak. At pH 5 and pH 6, BSA is marginally affected by the adsorption interaction, and below pH 5, the DOPE film becomes disordered, so there is a strong repulsive force interaction between the BSA and DOPE.

## Abstract 2

Nanopore technology was introduced for the study of the interaction between bovine serum albumin (BSA) and 1,2-dioleoyl-*sn*-glycero-3-phosphoethanolamine (DOPE) phospholipids.

## Graphical abstract

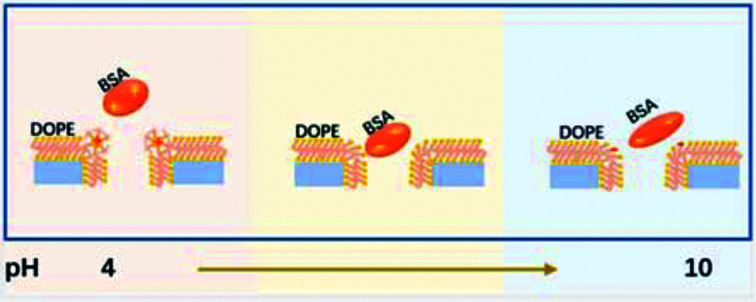

## Introduction

In recent years, liposomes have received increasing attention in developing efficient drug and pharmaceutical delivery systems.^1^ 1,2-dioleoyl-*sn*-glycero-3-phosphorethanolamine (DOPE) is an important component of liposome carriers. DOPE greatly affects the liposomes' activities *in vivo* due to its neutral property.^2^ Therefore, numerous research studies have focused on the interaction between plasma constituents and liposomes in order to improve the bioavailability of drugs. In the last few years, researchers have developed a variety of protein–lipid interaction characterization methods based on surface measurements, such as surface pressure, electrical potential and microscopic visualization measurements.^3–7^ However, all of the aforementioned methods are limited for the study of the interplay between lipids and proteins *in situ*. Nanopore technology is a promising approach for the study of DNA unzipping kinetics, DNA–protein interactions, and the dynamics of proteins.^8–17^ The investigation of the dynamics of these biopolymers is based on their interactions with the nanopore walls. Bovine serum albumin (BSA) is one of the main proteins in bovine serum; it belongs to the globular proteins and has great conformational adaptability. Therefore, in this work, we introduce nanopore technology for the study of the interaction between BSA and DOPE.

**Scheme 1:**
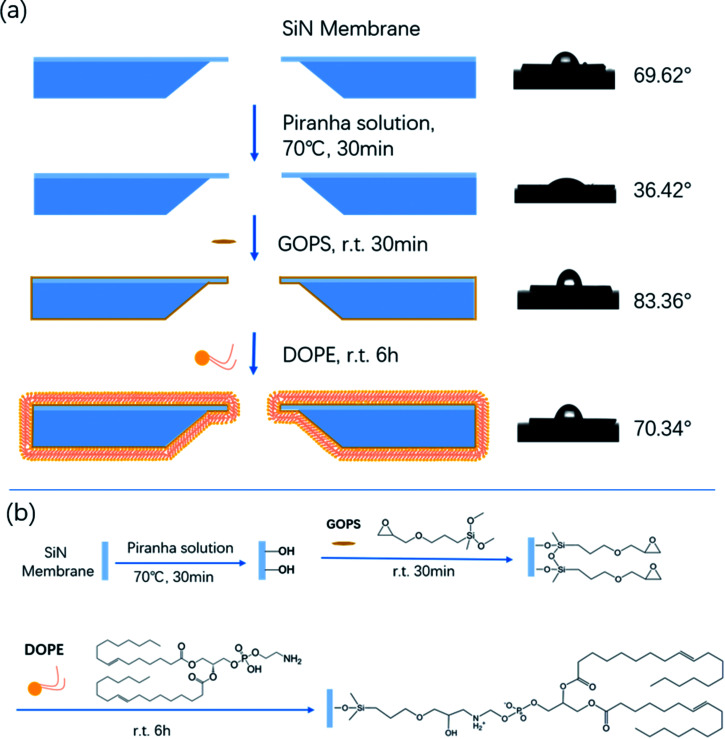
(a) Schematic of the chemical modification steps of the SiN_*x*_ nanopore with DOPE (left), and the film hydrophilicity test (right). (b) The reaction mechanism of the chemical modification.

To date, there are a variety of approaches using direct physisorption of lipids onto solid supports.^18,19^ However, the interface between the lipid membrane and solid substrate is not well-controlled, and the lifetime of non-covalently attached lipids is limited.^20^ In this report, we firstly develop a simple feasible bottom-up approach to assemble a tethered lipid layer (TLL) on the nanopore surface, and the modification steps are shown in Scheme 1a (left). The applied nanopores were fabricated *via* electric pulse breakdown. The organosilane GOPS directly self-assembles on the nanopore surface to form an epoxy group-coated layer for further reaction. The terminal epoxy groups were used to covalently attach the amino group on DOPE *via* an epoxy–amine reaction to form the TLL modified SiN_*x*_ nanopore, as shown in Scheme 1b.

## Results and discussion

The film hydrophilicity, thickness, and chemical composition for each modification step of the SiN_*x*_ substrate were characterized with contact angle measurements and X-ray photoelectron spectroscopy (XPS). As shown in Scheme 1a (right), the film hydrophilicity apparently changed after each modification treatment for the substrate. The contact angle of the uncoated SiN_*x*_ nanopore was found to be 36.42° after Piranha treatment, while it was 69.62° after the modification with DOPE. This suggests that the introduction of a lipid layer on the substrate contributes to the increase of the hydrophobicity of the film. XPS measurements were performed to verify the chemical composition of the coated film (Fig. S1†). In Table S1,† the element percentages of the bare (Piranha-treated) SiN_*x*_, GOPS-coated film, and DOPE film are compared. The SiN_*x*_ exhibits strong signals for O after coating with GOPS, which was attributed to the terminal epoxy group, and the DOPE film displays a high signal for C, which arises from the alkyl groups.

However, these characterization techniques described above cannot be used to probe the coating inside a nanopore. The ion flux through the nanopores is extremely sensitive to the nanopore coating thickness, since the ionic conductance (*G*) depends quantitatively on the nanopore diameter (*d*).

The effective diameter was estimated based on the empirical formula:^21,22^

1 *G* = *σ*[(4*L*/π*d*^2^) + (1/*d*)]^−1^

**Fig. 1:**
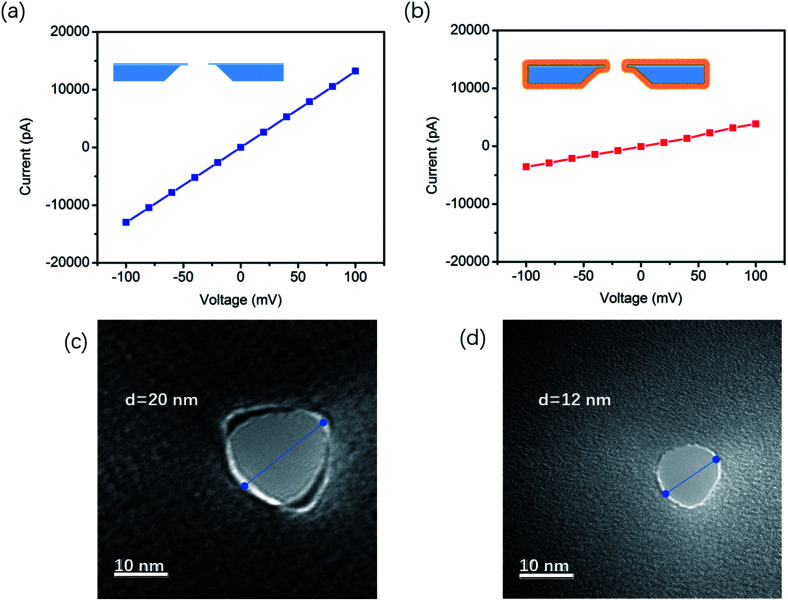
*I*–*V* curves of unmodified (a) and DOPE-modified (b) SiN_*x*_ nanopores in 1 M KCl (pH 7); TEM images of an unmodified SiN_*x*_ chip with a nanopore of 20 nm diameter (c) and a DOPE-modified SiN_*x*_ chip with a nanopore of 12 nm diameter (d).

Herein, *σ* is the electrical conductivity (1 M KCl, pH 7, 10.6 S m^−1^), and *L* is the length of the nanopore. In order to testify this, the ion-conductance of the bare and modified nanopores was measured (Fig. 1a and b) on a patch clamp. In terms of the size of the TLB modified nanopore, *d*′ = *d*_bare_ − 2*δ*, where *d*_bare_ is the calculated diameter of the uncoated nanopore and *δ* is the thickness of the DOPE film. According to a literature report, the thickness of the DOPE layer is about 3.6 nm.^18,23^ The length of the modified nanopore is increased by the lipid layer coating, *L*′ = *L*_bare_ + 2*δ*, where *L*_bare_ is the SiN_*x*_ thickness (20 nm in this work). The calculated diameters of the bare SiN_*x*_ nanopore and the TLL-modified nanopore are 20 nm and 13 nm respectively, which agrees very well with the physical sizes measured by the TEM technique (Fig. 1c and d).

**Fig. 2:**
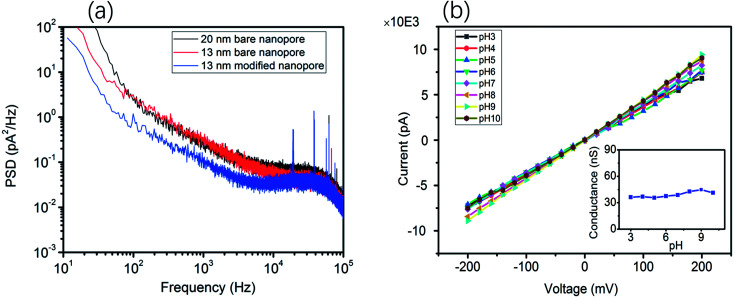
(a) Power spectrum densities (PSDs) of the ionic current baseline for the bare nanopore of 20 nm diameter (black), the bare nanopore of 13 nm diameter (red) and the TLL-modified (blue) nanopore of 13 nm diameter at 100 mV with 1 M KCl (pH 7). (b) *I*–*V* curves of the TLL-modified nanopore of 13 nm diameter at various pH values (from 3 to 10) in 1 M KCl buffer solution; the inset is the nanopore conductance as a function of pH for the modified nanopore.

Moreover, the ionic current noise between the bare nanopore and the TLL-modified nanopore was compared. The power spectrum densities (PSDs) were obtained from the 10 second baseline. As shown in Fig. 2a, the 1/*f* noise has been depressed after the modification, which is favorable for recording the translocation signature. In addition, the *I*–*V* curves were recorded to testify the stability of the TLL nanopore with a broad range of pH values (from 3 to 10) in 1 M KCl (Fig. 2).

This result indicates that the conductance of the TLL-modified nanopore does not change significantly under these conditions (Fig. 2, inset). In order to explore the potential applications of the protein adsorption interaction using the TLL-modified nanopore, we carried out measurements at distinct pH values for BSA. The ionic current traces of 0.5 μM BSA translocation were recorded in 1 M KCl (10 mM Tris, 1 mM EDTA) buffer at various pH values with a modified nanopore of 13 nm diameter at 100 mV.

**Fig. 3:**
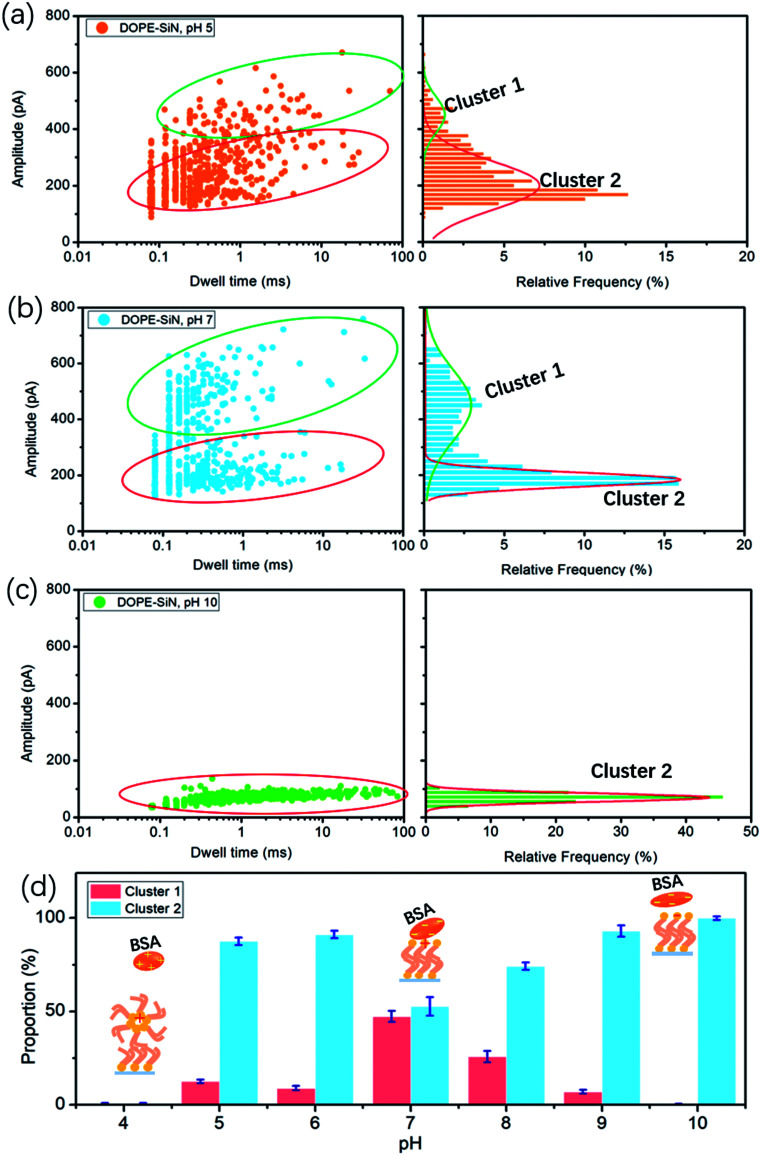
Event scatter plots for the translocation of 0.5 μM BSA through a 13 nm TLL-modified nanopore under 100 mV at pH 5 (a), pH 7 (b) and pH 10 (c). Amplitude histograms are fitted with Gaussian distributions. (d) The proportion of cluster 1 and cluster 2 at different pH values (from 4–10).

According to the reported data, the isoelectric point (PI) of BSA ranges from pH 5.1 to pH 5.5.^15^ Hence, BSA is negatively-charged above pH 5, and electrically-neutral at pH 5. The cumulative ionic current trace results are presented in event distribution plots (Fig. 3). The ionic current was measured at pH 5, which is close to the isoelectric point of BSA. Fig. 3a shows that there are two clusters of BSA blockage events: the current drop (Δ*I*) of cluster 1 is mainly between 400 and 600 pA, and the Δ*I* of cluster 2 is 100–300 pA. At pH 7, there are also two clusters (Fig. 3b). Compared with the results at pH 5, the percentage of cluster 2 became higher and the blockage time became shorter (Fig. 3d).

Previous studies have demonstrated that the current drop is approximately proportional to the excluded volume (*V*) of the translocating particle inside the nanopore and can be approximately described as:^24^

2 Δ*I* = (*σψ*/*L*_eff_^2^)*V*

In this formula, *σ* is the conductivity of the solution (1 M KCl, pH 7, 10.6 S m^−1^), *ψ* is the applied voltage, and *L*_eff_ is the effective length of the nanopore (∼27 nm in this work). It is reported that the volume of BSA is about 224 nm^3^. Therefore, according to formula (2), the calculated Δ*I* of BSA in this experiment is 326 pA. Previous studies have shown that to compare long thin rods with spheres and short rods of the same volume, for the spheres and short rods Δ*I* must be multiplied by 3/2 for the values to agree on an absolute basis.^25^ Thus, the value of Δ*I* after absolute calibration is close to 490 pA. This suggests that cluster 1 events are attributed to BSA molecules translocating through the nanopores, and cluster 2 events are protein molecules that failed to pass through. At pH 5 the electric force of BSA in the nanopore is lower than that of BSA at pH 7, which causes more molecules to fail to pass through the nanopore at pH 5.

Earlier research shows that the volume of BSA molecules is expanded at low pH values.^26^ Based on formula (2), Δ*I* increases with the increase of the volume. However, we couldn't get any current blockage signal below pH 5. According to a previous study,^27^ DOPE lipid easily forms a hexagonal (HII) phase at pH values below 5. The hexagonal phase is considered to exhibit a ‘cone’ shape, where the polar headgroup region is at the smaller end of the cone.^24^ The alkyl groups of DOPE are exposed outside, and the DOPE film and BSA become positively-charged at pH values below 5. As a result, the repulsive fore between BSA and the disordered DOPE film hinders BSA from moving to the interface, such that it is hard to get a nanopore translocation event.

At pH 10, far away from PI, there is only one cluster, as presented in Fig. 3c, and the current drop becomes very short (only around 100 pA), but the blockage time is obviously longer than that under neutral and acidic solution conditions. That is to say, barely any BSA proteins successfully translocate through the TLL-modified nanopore. Exposure to alkali will cause BSA dimerization, unfolding, and eventual aggregation. The hydrophobic interaction between BSA and DOPE becomes weak due to the few hydrophobic groups of BSA become exposed. DOPE is gradually changed to negatively-charged, and from pH 8 to 10 the proportion of cluster 1 becomes less and less (Fig. S2a and b†). This illustrates that, in alkali solution, the electrostatic repulsion between BSA and DOPE becomes the main interaction, which influences the BSA translocation through the modified nanopore.

## Experimental

### Materials and methods

#### Materials

The SiN_*x*_ membranes were purchased from Norcada (NT001Y), with a thickness of 20 nm and a window size of 10 μm × 10 μm. Anhydrous methanol was acquired from Aldrich. 3-Glycidyloxypropyltrimethoxysilane (GOPS), 1,2-dioleoyl-*sn*-glycero-3-phosphoethanolamine (DOPE), potassium chloride (KCl), ethylenediaminetetraacetic acid (EDTA) and bovine serum albumin (BSA) were purchased from Sigma. Deionized water was obtained from a molecular H_2_O system. The buffer solution was filtered with a 0.22 μm filter before use.

#### Ionic transport through the nanopore

The prepared nanopore chip was embedded in a two-chamber cell where both sides of the nanopore were separated. Each side was filled with 1 M KCl, 10 mM Tris–HCl, and 1 mM EDTA buffer at different pH values (from pH 3 to pH 10). Two Ag/AgCl electrodes were inserted into each chamber. A bias voltage was applied by a patch clamp amplifier (Axon 200B). The ion current blockade measurements were performed at 8-pole and 40 kHz with pClamp 10 software. All of the demonstrated events in the figures have been filtered by a 10 kHz low-pass Bessel filter for clarity.

#### BSA translocation through the nanopore

The prepared nanopore chip was embedded in a two-chamber cell where both sides of the nanopore were separated. Each side was filled with 1 M KCl, 10 mM Tris–HCl, and 1 mM EDTA buffer at different pH values (from pH 5 to pH 10). BSA solution was diluted to 0.5 μM. Two Ag/AgCl electrodes were inserted into each chamber. A bias voltage was applied by a patch clamp amplifier (Axon 200B). The ion current blockade measurements were performed at 8-pole and 40 kHz with pClamp 10 software. All of the demonstrated events in the figures have been filtered by a 10 kHz low-pass Bessel filter for clarity.

### Nanopore fabrication and modification

The nanopores were fabricated with SiN_*x*_ membranes (thickness 20 nm) *via* electric pulse breakdown, which was performed on Keithley 2450 equipment. The diameter of the obtained nanopores is about 20 nm. First, the freshly prepared nanopores were treated with Piranha solution (volume ratio 1 : 3 H_2_O_2_ : H_2_SO_4_) for 30 min at 70 °C, followed by rinsing with deionized water to clean the nanopores and simultaneously introduce hydroxyl groups onto the interior nanopore surface. Then, the chip was soaked in 0.000005% GOPS anhydrous methylbenzene solution for 30 min, followed by washing with methylbenzene, acetone, isopropanol, and deionized water to remove the residual GOPS silane on the pore surface. After forming epoxy groups on the surface, the chips were treated with 0.001 M DOPE for 6 hours and then washed with chloroform and alcohol.

### Characterization

Contact angle (DSA100) equipment was used for the detection of the hydrophilicity of the films. A spectroscopic ellipsometer (Gaertner L116) was used to characterize the film thickness. An XPS instrument (Escalab 250Xi) equipped with a monochromatic Al-Kα source was used to analyze the chemical components of the films.

## Conclusions

In this study, we introduce nanopore technology to investigate the interaction between BSA and lipid films based on a TLL-modified solid-state nanopore. By analyzing BSA translocation at different pH values, these results reveal that the interaction between DOPE and BSA is affected by the pH, as shown in Fig. 3d.

(i) Far above the PI (pH > 7), a weaker hydrophobic interaction and stronger electrostatic repulsion exist between the DOPE and BSA molecules, so a lower blockage current and longer blockage time for BSA are observed.

(ii) At pH = 7, the BSA structure nearly does not change and the highest current is detected.

(iii) BSA carries nearly zero net charge at pH 5 and pH 6 (Fig. S2c†), and is marginally affected by the adsorption interaction.

(iv) At pH values below 5, the DOPE film becomes disordered, and no translocation events are observed due to the strong repulsive force interaction between BSA and DOPE.

## Conflicts of interest

There are no conflicts to declare.

## Supplementary Material

RA-009-C9RA00698B-s001
