# Supplementary material for: Investigation of the adsorption behavior of BSA with tethered lipid layer-modified solid-state nanopores in a wide pH range
Source: RSC Adv. 2019 May 17;9(27):15431–6. doi: 10.1039/c9ra00698b (PMC9064230; doi:10.1039/c9ra00698b)
Supplement: RA-009-C9RA00698B-s001 [file RA-009-C9RA00698B-s001.pdf]

Electronic Supplementary Material (ESI) for RSC Advances.
This journal is © The Royal Society of Chemistry 2019

Electronic Supplementary Information

Investigation of adsorption behavior of BSA with tethered lipid layer modified solid-state nanopores in a wide pH range

Haibing Tian,[a,b†] Wanyi Xie,[a,b†] Shixuan He,[a] Daming Zhou,[a] Shaoxi Fang,[a] Liyuan Liang[a,b*] and Deqiang Wang[a,b*]

1. *XPS measurements*

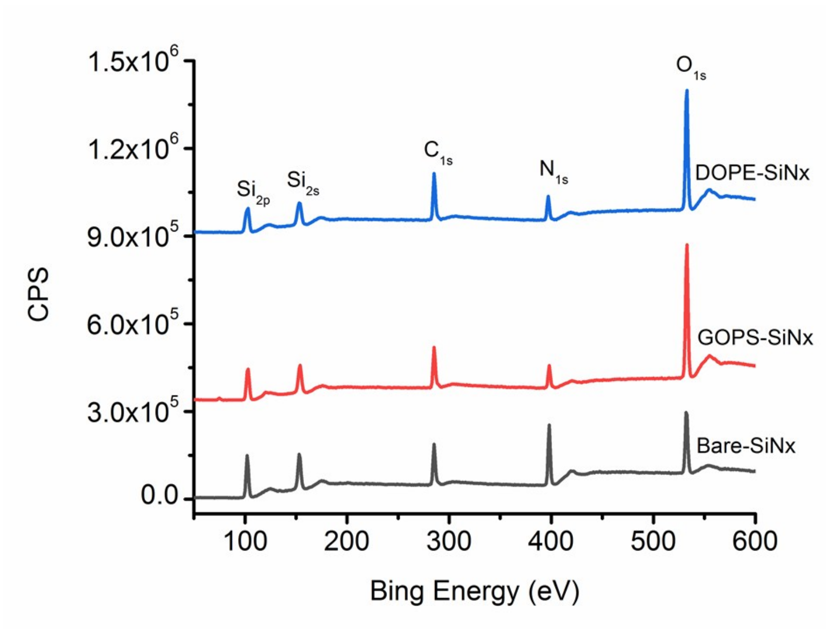

Figure S1. XPS spectra of bare-SiNx (A1), GOPS coated SiNx (A2), DOPE modified SiN (A3)

Table S1. characterization of atom percentage for different modification steps.

Modification steps	Si2p (%)	C1s (%)	N1s (%)	O1s (%)	P2p (%)
Bare-SiN_x	30.37	27.05	23.96	18.19	0.49
GOPS-SiN_x	25.79	27.65	8.29	37.4	0.23
DOPE-SiN_x	25.01	32	10.16	32.82	---

2. *BSA translocation through nanopore at different pH* conditions

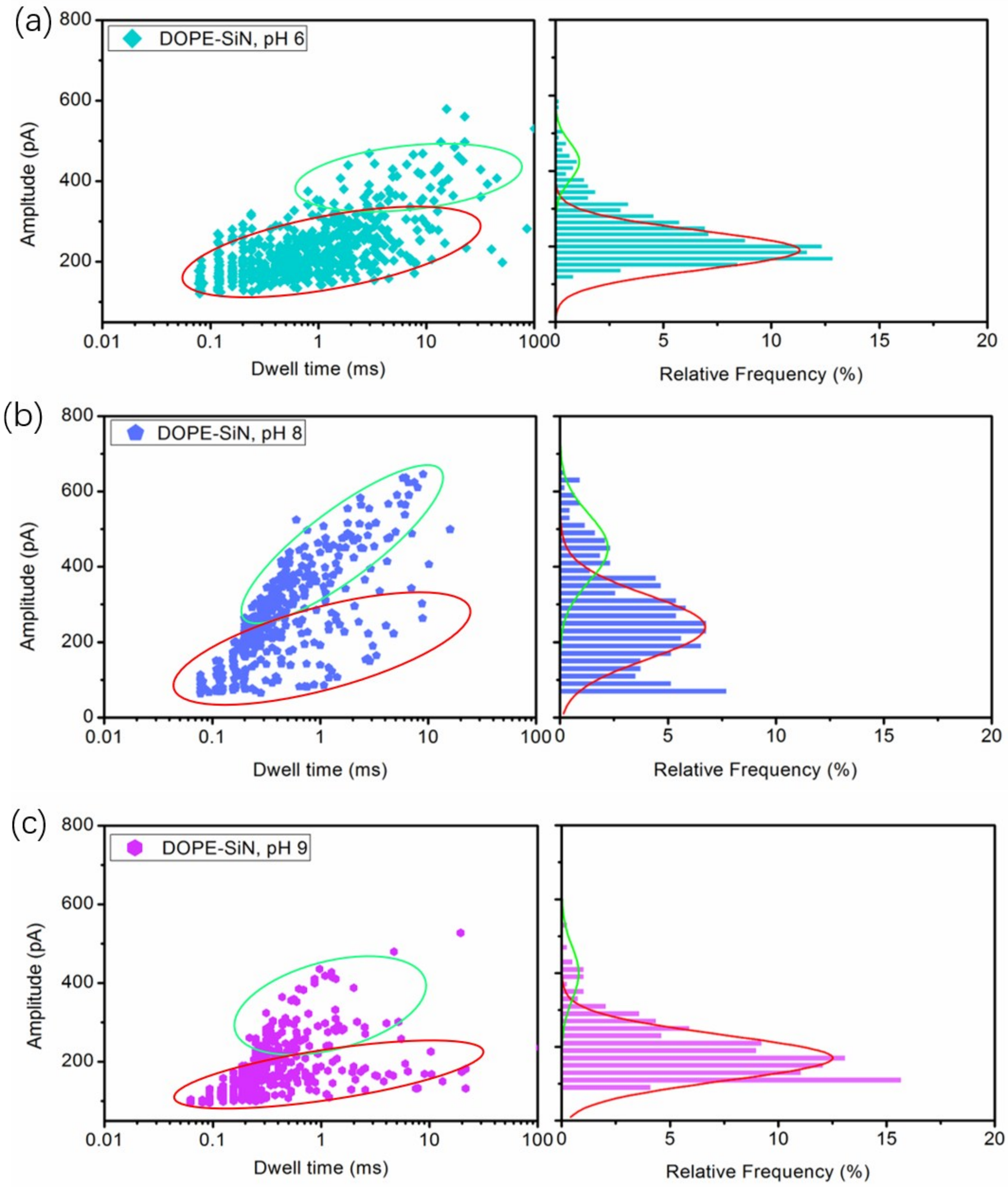

Figure S2. Event scatter plots for translocation of 0.5 μM BSA through a 13 nm TLL-modified nanopore under 100 mV at pH 6 (a), pH 8 (b) and pH 9 (c), respectively. Amplitude histograms are fitted with Gaussian distributions
